# CD8 + T-cell marker genes reveal different immune subtypes of oral lichen planus by integrating single-cell RNA-seq and bulk RNA-sequencing

**DOI:** 10.1186/s12903-023-03138-0

**Published:** 2023-07-08

**Authors:** Jinhao Zhang, Gaoge Peng, Hao Chi, Jinyan Yang, Xixi Xie, Guobin Song, Lisa Jia Tran, Zhijia Xia, Gang Tian

**Affiliations:** 1 School of Stomatology, Southwest Medical University, Luzhou, China; 2 Clinical Medical College, Southwest Medical University, Luzhou, China; 3 Department of General, Visceral, and Transplant Surgery, Ludwig-Maximilians-University Munich, Munich, Germany; 4 Department of Laboratory Medicine, The Affiliated Hospital of Southwest Medical University, Luzhou, China

**Keywords:** Autoimmunity, OLP, CD8 + T cells, Unsupervised hierarchical clustering, Single-cell sequencing

## Abstract

**Background:**

Oral lichen planus (OLP) is a local autoimmune disease induced by T-cell dysfunction that frequently affects middle-aged or elderly people, with a higher prevalence in women. CD8 + T cells, also known as killer T cells, play an important role in the progression and persistence of OLP. In order to identify different OLP subtypes associated with CD8 + T cell pathogenesis, consensus clustering was used.

**Methods:**

In this study, we preprocessed and downscaled the OLP single-cell dataset GSE211630 cohort downloaded from Gene Expression Omnibus (GEO) to finally obtain the marker genes of CD8 + T cells. Based on the expression of marker genes, we classified OLP patients into CMGs subtypes using unsupervised clustering analysis. The gene expression profiles were analyzed by WGCNA using the “WGCNA” R package based on the clinical disease traits and typing results, and 108 CD8 + T-cell related OLP pathogenicity-related genes were obtained from the intersection. Patients were once again classified into gene subtypes based on intersection gene expression using unsupervised clustering analysis.

**Results:**

After obtaining the intersecting genes of CD8 + T cells related to pathogenesis, OLP patients can be precisely classified into two different subtypes based on unsupervised clustering analysis, and subtype B has better immune infiltration results, providing clinicians with a reference for personalized treatment.

**Conclusions:**

Classification of OLP into different subtypes improve our current understanding of the underlying pathogenesis of OLP and provides new insights for future studies.

**Supplementary Information:**

The online version contains supplementary material available at 10.1186/s12903-023-03138-0.

## Introduction

The OLP is an autoimmune disease caused by dysfunctional T cells, with lesions often found in the buccal mucosa, tongue, and gums [1]. The prevalence is estimated to be 0.5–2.0% [2, 3], and OLP frequently affects middle-aged or older adults, with a higher prevalence in women [4, 5]. The most common forms are reticular, vesicular, papular, and plaque, while atrophic and maculopapular forms are less common. Clinically OLP may occur individually or in various combinations [6]. There are several common symptoms of OLP, including burning sensations and chronic pain, but lesions of the reticular and papular regions usually present without symptoms. However, atrophic and erosive forms of OLP can negatively affect patients’ quality of life, causing sensitivity, burning symptoms and discomfort [7]. A common feature of OLP is immune infiltration, particularly CD8 + lymphocytes, as well as the expression of Th1 and Th2 cytokines in OLP lesions and tissue secretions [8] The complex cytokine network is thought to play an essential role in the progression and persistence of OLP [9], so there is a speculation on the pathogenesis: infiltrative cytotoxic CD8 + T cells promote apoptosis of oral mucosal basal cells, leading to autoimmunity. keratin-forming cells replace CD8 + T cells in the major tissue, which is activated directly by the major histocompatibility complex (MHC)-1 on keratinocytes or indirectly by antigen-presenting [10]. OLP rarely regresses spontaneously and is classified as a potentially malignant disease by the World Health Organization[11]. Therefore, exploring the malignant mechanism of OLP to OSCC is crucial for early diagnosis of OSCC and for providing more effective therapeutic measures.

In dentistry and oral pathology, OLP is one of the most frequently encountered mucosal diseases. OLP does not have a completely curative treatment available[12]. However, recent developments in microarray and RNA sequencing technologies have ushered in a new shift in biomedical research. In the present study, we screened highly variable genes through a series of analyses of single-cell sequencing data and transcriptome data based on various databases to identify potential regulatory mechanisms of OLP and potential biomarkers in the pathogenesis of OLP.

## Method

### Raw data collection

The single-cell data GSE211630 cohort containing 10× scRNA-seq data from five OLP samples and one normal oral mucosa sample were downloaded from the GEO database. For gene expression profile data, two cohorts GSE38616 and GSE52130 were merged and batch effects [13] were removed from the GEO database.

### Data processing and analysis of scRNA-seq

The 10× scRNA-seq data were processed as follows: (1) 10× scRNA-seq data were converted to Seurat objects using the R software “Seurat” package [14]; (2) Counts were quality controlled by excluding low-quality cells based on mitochondrial or ribosomal gene percentages (Quality Control(QC)); (3) Screening the first 2000 highly variable genes after QC using the “FindVariableFeatures” function; (4) Downscaling and cluster identification were performed using principal component analysis (PCA) based on 2000 genes and unified flow approximation and projection (Uniform Manifold Approximation and Projection (UMAP)) [15]; (5) Using the “Find All Markers” function, identified significant marker genes within different clusters by setting log2FC to 0.3 and min. pct to 0.25; (6) Our analysis of cluster annotation was conducted using the “SingleR” package [16] in R software. Next, Fisher precision tests were performed to identify potentially significant cell types. We calculated FC values for each cell type in tumor and normal samples and identified cell types with FC > 4 or FC < 0.25, with P < 0.05 as the key cell type. In addition, we performed functional enrichment analysis of the identified central cell types using the R software “ReactomeGSA” package [17]. Our enrichment analysis was conducted by using the “analyse_sc_clusters” function and the “pathways” function to extract the results. The single-cell dataset was scored using the R software “irGSEA” package, differentiation trajectory inference was done using Monocle 3 and CytoTRACE, and dimensionality reduction was performed using the “DDRTree” method [18, 19]. A statistical technique called “BEAM” was then used to calculate the contribution of genes to cell development, and the top 100 genes were then visualized.The “patchwork” package was used for intercellular communication analysis and network visualization.

### Identification of CMGs

Find differentially expressed genes using the FindAllMarkers function, which calculated the average expression of each gene for each subpopulation and evaluated whether genes were differentially expressed between subpopulations using the Wilcoxon Rank Sum test.

### Weighted gene co-expression network analysis (WGCNA)

WGCNA was performed using the R package of “WGCNA” (version 1,70.3) [20] to identify co-expression modules). During the subsequent WGCNA analysis, the top 25% of genes with the highest variance were used to ensure quality. Our weighted adjacency matrix (WAM) was constructed via a soft thresholding power we had recommended and then transformed into a topological overlap matrix (TOM). By setting a minimum module size of 200, we calculated modules using a TOM dissimilarity measure (1-TOM) based on hierarchical clustering trees. Each module was assigned a random color. Every module’s eigengene profile represented global gene expression.

Module Significance (MS), a measure of the relationship between modules and disease states, and Gene Significance (GS), a measure of the correlation between genes and clinical traits, were discussed.

### Unsupervised clustering of OLP patients

With 11 CMGs expression profiles, we used an unsupervised clustering algorithm(“ConsensusClusterPlus” R package) [21] with 1,000 iterations to classify 14 OLP samples(Including samples GSM946260, GSM946261, GSM946262, GSM946263, GSM946264, GSM946265, GSM946266 of dataset GSE38616 and GSM260095, GSM260096, GSM260097, GSM260098, GSM260099, GSM260100, GSM260101 of dataset GSE52130)into 2 clusters. According to a combination of cumulative distribution function (CDF) curves, consensus matrices, and consistent clustering scores (> 0.9), we evaluated the optimal number of clusters using the maximum number of subtypes k (k = 9). In the same way, we performed a second clustering of OLP samples with 108 genes related to CD8 + T cells involved in OLP pathogenesis.

### Functional enrichment analysis of mechanism-associated genes

Gene ontology (GO) and KEGG(www.kegg.jp/kegg/kegg1.html) [22–24] enrichment analysis of the genes contained in the module were carried out using the R package “clusterProfiler”. Significantly enriched functions or pathways were identified according to the criteria: adjusted *P* < 0.05. We used the single sample gene set enrichment analysis (ssGSEA) method to assess the relative abundance of infiltrating immune cells in a subpopulation of OLP patients.

### Construction of the CMGs score

Using PCA, we developed a CMGs scoring scheme to quantify the level of genetic modification in individual patients. PCA was then conducted on the expression profiles of prognostic differential expressed genes (DEGs), and the principal components PC 1 and PC 2 were extracted as characteristic scores. The CMGs score was defined by previous studies as follows: CMGs score = (PC1i + PC2i), where “i” is the expression of genes associated with the involvement of CD8 + T cells in the pathogenesis of OLP [25].

### Statistical analysis

Statistical analysis was performed using R software v4.2.1. The correlation between genes and immune cell infiltration was assessed using Spearman correlation analysis. Both groups were compared using the Wilcox test to determine the proportion of infiltrating immune cells. Statistical significance was determined by a *P* < 0.05 and a false discovery rate (FDR) < 0.05 was considered significant.

## Result

### Cell clustering and annotation of scRNA-Seq for normal and OLP samples

**Fig. 1:**
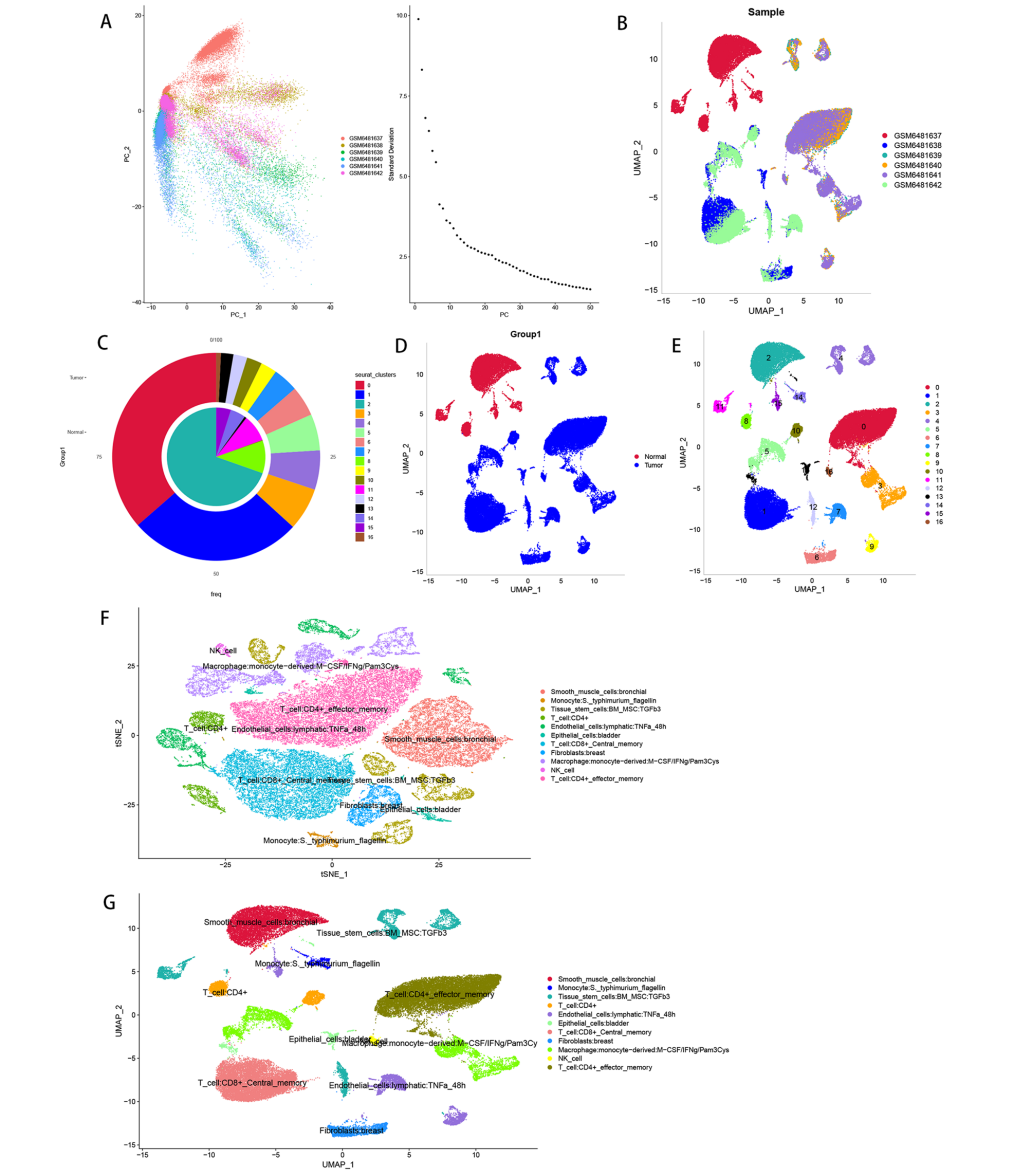
Single-cell data preprocessing. (**A**) PCA downscaling analysis. (**B**) Cell distribution maps of six tissue samples. (**C-E**) Distribution of normal group disease group and subpopulation distribution of cells. (**F-G**) Single-cell annotation results

First, single-cell data were preprocessed. Supplementary Fig. 1A is a control chart before and after single-cell quality control. Supplementary Fig. 1B illustrates the 20 most highly variant genes. We used the scaledata function to scale the selected high-variant genes and found the anchor points by PCA downscaling. Then, we selected the data of the top 15 PCs for downscaling. (Fig. 1A-B) The results of different sample sets visualized using umap are shown in the figure (Fig. 1C), where GSM6481637 is normal tissue and the remaining 5 are tissues from patients with lichen planus. Afterward, we used the findclusters function of the “seuret” package to divide the cells into 16 subpopulations and calculated the abundance of these 16 subpopulations in patients and normal tissues (Fig. 1D). The distribution of cells in patient and normal tissues is shown in Fig. 1E, shows the distribution of 16 cell subpopulations. Next, we annotated the sample cells with SingleR and visualized them with tSNE and UMAP downscaling, respectively, and finally identified different clusters with a total of 11 cells, including B cell: Plasma_cell; NK_cell; NK_cell: CD56hiCD62L+; Immune cells: CD4+_central_memory; Immune cells: CD4+_effector_memory; Immune cells: CD8+; Immune cells: CD8+_Central_memory; Immune cells: CE’8+_effector_memory; Immune cells: CD8+_effector_memory_RA; Immune cells: CD8+_naive; Immune cells: Treg: Naïve(Fig. 1F-G).

### Cell developmental trajectory and cell communication analysis

**Fig. 2:**
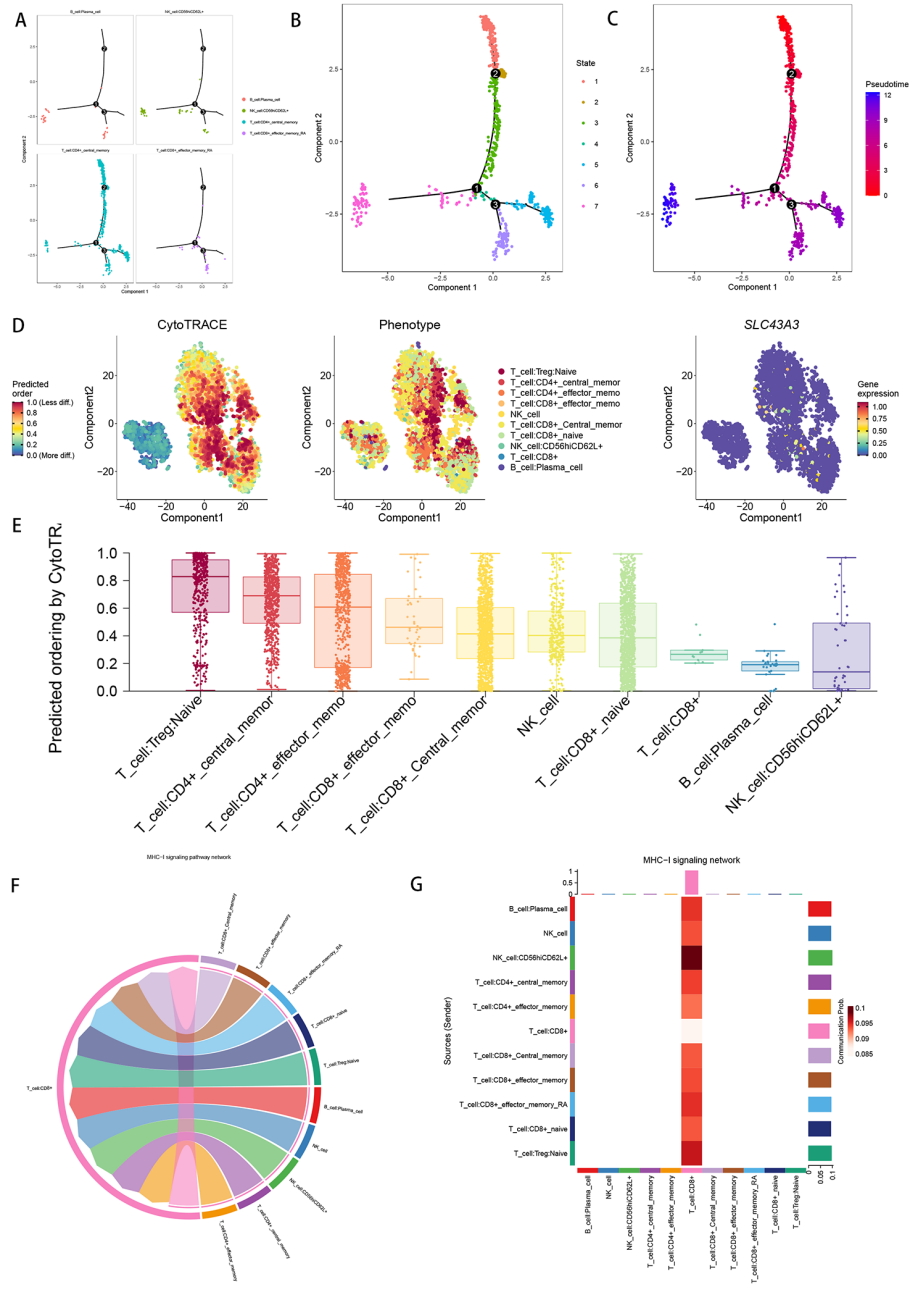
Analysis of cellular communication and cell trajectories. (**A-C**) Cell trajectory and pseudo-time analysis. (**D-E**) CytoTRACE was used to assess the differentiation potential of individual cell subpopulations. (**F-G**) Cellular communication networks identify MHC-I as playing a key role in the communication network

In order to analyze the trajectories and pseudotimes of the four important cell types, we used the “monocle” package. We observed that plasma cells and NK cells both correspond to states 3, 4, 6, and 7, while CD4 + central memory T cells appeared in the entire state (Fig. 2A-C). To visualize the top 100 genes during cell development, we calculated the contribution of genes during cell differentiation (Supplementary Fig. 3B). The intercompartmental communication network was investigated by calculating communication probabilities (Supplementary Fig. 3A). We then used CytoTRACE for cellular assessment of cell subpopulation differentiation potential (Fig. 2D-E) and found that CD4 + central memory T cells had the highest CytoTRAC score, followed by CD8 + effector memory T cells, from which we inferred that the proposed temporal direction should be from CD4 + central memory T cells to CD8 + effector memory T cells, and then to plasma cells and NK cells.

Additionally, ligand-receptor networks and specific pathways were used to infer cell-cell communication networks. According to our study, MHC-I was crucial to the communication network, which also validated the importance of CD8 + T cells as our target cell population. (Fig. 2F-G).

### Functional pathway enrichment of each cell subpopulation

**Fig. 3:**
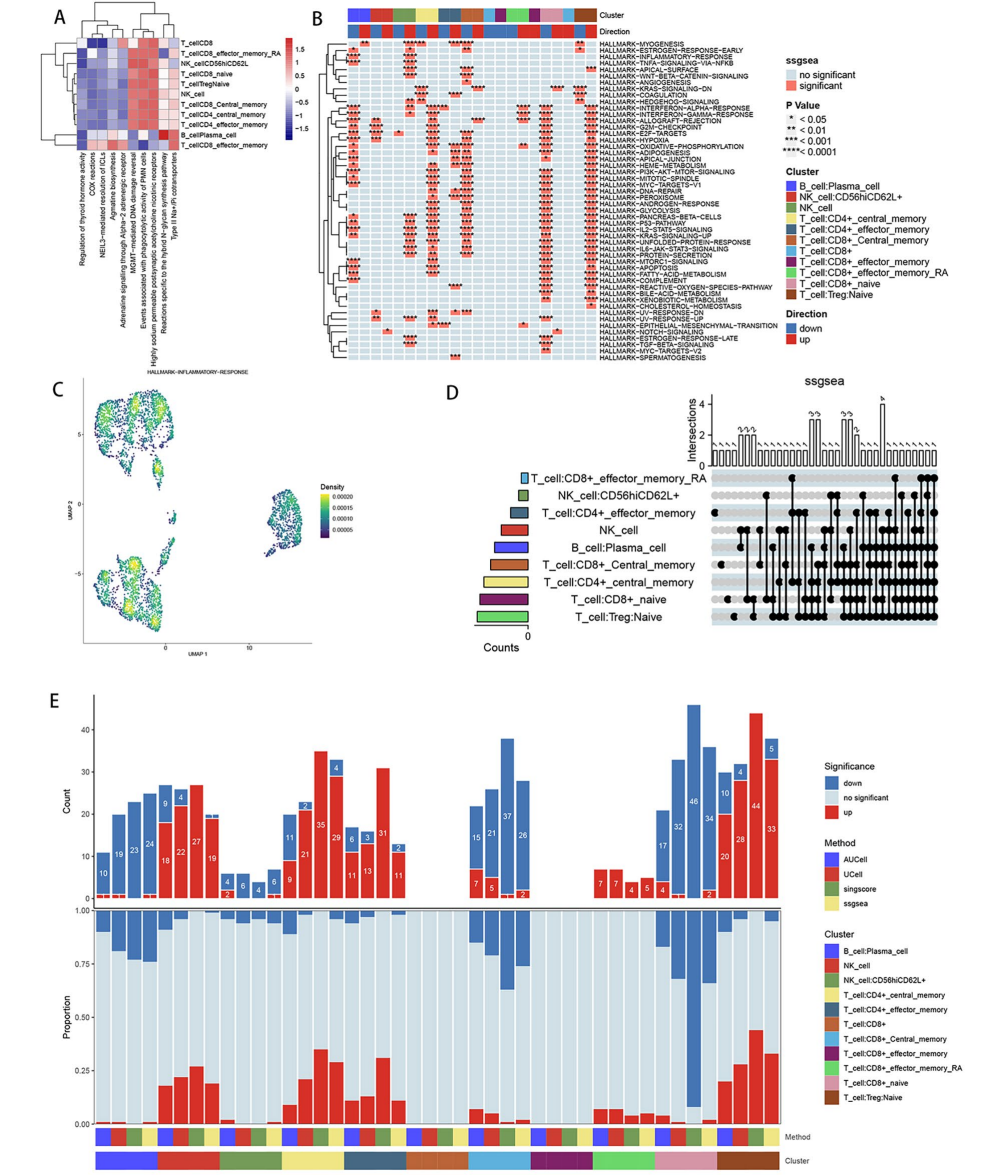
Functional enrichment. (**A**) Functional enrichment analysis for the identified hub cell types using the “ReactomeGSA” package. (**B-E**) Functional enrichment analysis for the identified hub cell types using the “irGSEA” package

ReactomeGSA functional enrichment analysis showed that three pathways, MGMT-mediated DNA damage reversal, events associated with phagocytic activity in PMN cells, and high sodium-permeable postsynaptic acetylcholine nicotinic receptors, were highly expressed in most cell types (Fig. 3A). Using the “irGSEA” package, we performed single-sample gene set enrichment analysis (ssGSEA). The heat map of differentially upregulated or differentially downregulated gene sets in the ssGSEA enrichment analysis (Fig. 3B) with the bars to the left of the upset plot demonstrates the number of gene sets with statistically significant differences for each cell subpopulation in the comprehensive assessment, and the bars above the upset plot represent the differential gene sets with intersections The bars above the upset plot represent the number of differential gene sets with intersections, and multiple dotted lines represent multiple cell subpopulations taking intersections (Fig. 3D). The density scatter plot combines the enrichment fraction of gene sets and the projection of cell subpopulations in low-dimensional space to demonstrate the spatial expression level of a specific gene set. Among them, the more yellow color represents a higher enrichment fraction, where naive CD8 + T cells, CD4 + central memory T cells, and CD8 + effector memory T cells have a higher enrichment fraction (Fig. 3C). Stacked bar graphs specifically show the number of up-regulated, down-regulated, and no statistically different gene sets in each cell subpopulation in four gene set enrichment analysis methods (AUcell, Ucell, singersore, ssgsea); the upper bars represent the number of genes differing in different methods in each subpopulation, with red representing up-regulated differential gene sets and blue representing down-regulated differential gene sets; the middle bar represents the proportion of up-regulated, down-regulated and no statistically significant gene sets in different methods in each subpopulation (Fig. 3E).

### Extraction of marker genes for target cell subpopulations

**Fig. 4:**
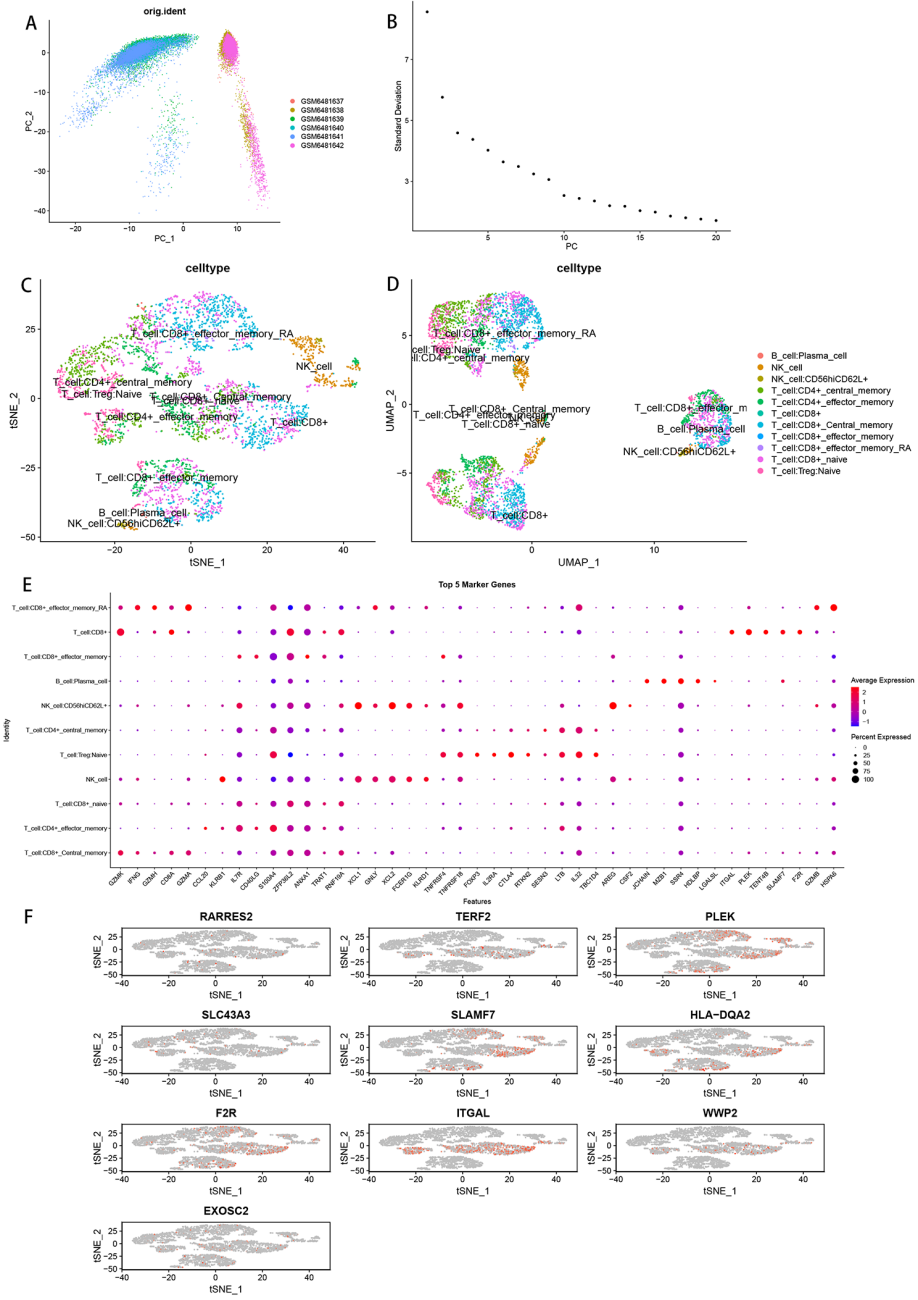
Extraction of target cell subsets. (**A**) Principal component analysis of immune cell subpopulations with highly variable genes. (**B**) Sorting of downscaled anchor points. (**C-D**) Distribution of results after annotation of immune cell subpopulations. (**E**) Expression of immune cell subpopulation Top5 Marker genes in different subpopulations. (**F**) Expression of marker genes in CD8 + T cells in immune cells

To further investigate the immune characteristics of OLP, we extracted immune cells from all samples for further analysis. We selected the first 8 PCs to downscale the data (Fig. 4A-B). Next, we used SingleR to annotate immune cell subpopulations after tSNE and UMAP downscaling, and a total of 11 cell subpopulations were identified, including B cell: Plasma_cell; NK_cell; NK_cell: CD56hiCD62L+; Immune cells: CD4+_central_memory; Immune cells: CD4+_effector_memory; Immune cells: CD8+; Immune cells: CD8+_Central_memory; Immune cells: CE’8+_effector_memory; Immune cells: CD8+_effector_memory_RA; Immune cells: CD8+_naive; Immune cells: Treg: Naïve (Fig. 4C-D). We identified 11 marker genes subgroups using the findallmarkers function and extracted the top 5 differentially expressed genes for display, with a logFC threshold of 0.3 (logFC threshold = 0.3 is considered differentially expressed and greater than this threshold) and min. pct selected to be 0.25 (when a gene is in two clusters (only when a gene is expressed in more than 25% of cells in one or both clusters (Fig. 4E), differential analysis was included). The expression of immune cell: CD8 + marker genes in immune cell subpopulations was subsequently visualized with tSNE. The results showed that PLEK, SLAMF7, F2R, and ITGAL were highly expressed in the immune cell: CD8 + subpopulation, while ITGAL was also expressed in the corresponding immune cell: CD4+_effector_memory (Fig. 4F).

### Identification of cellular subtypes

**Fig. 5:**
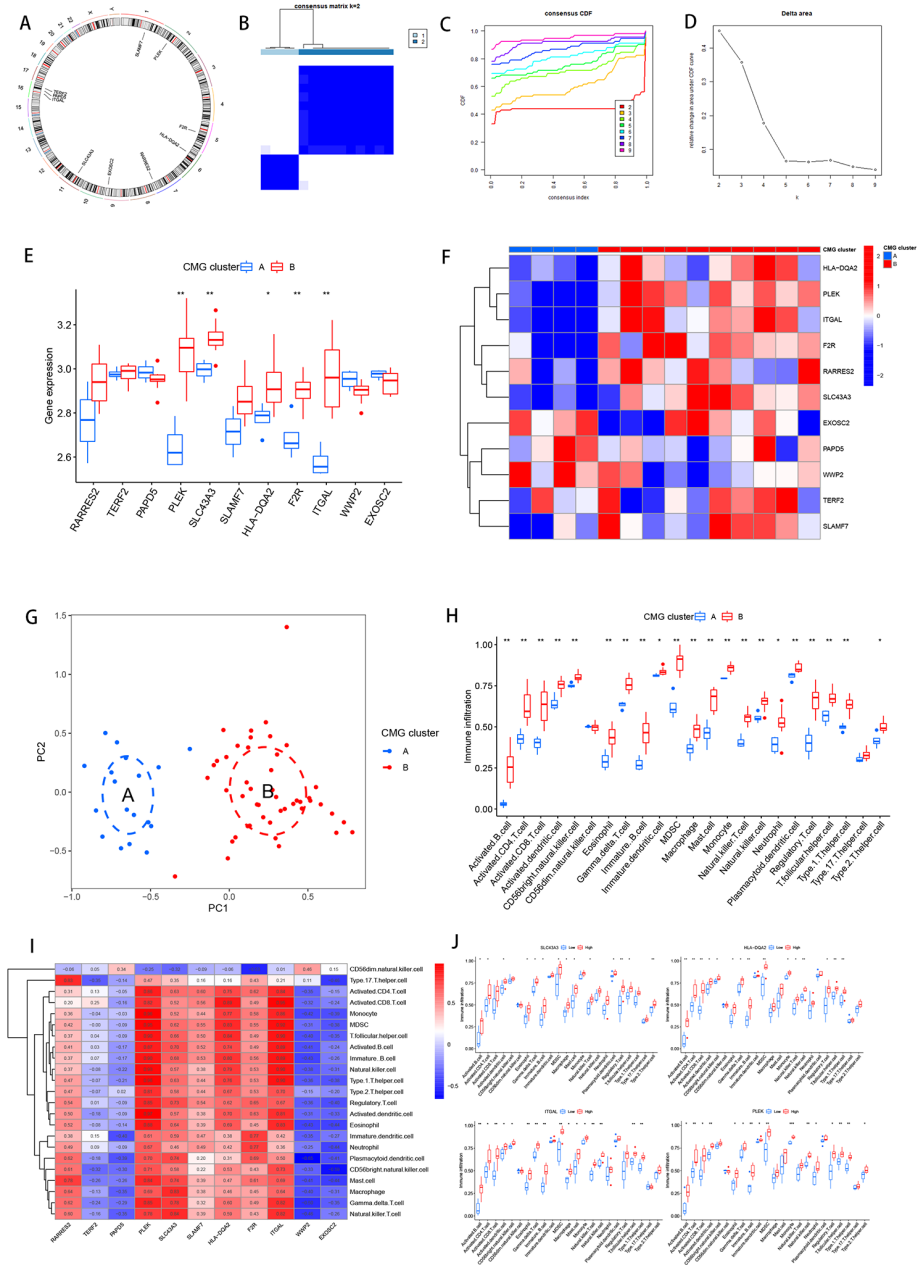
Consensus clustering identifies the molecular subtypes of marker genes. (**A**) Chromosomal circle diagram of 11 marker genes. (**B**) Consensus matrix for k = 2. (**C**) Consensus clustering CDF for k = 2 to 9. (**D**) Relative change in area under the cumulative CDF curve for k = 2 to 9. (**E**) Expression levels of marker genes in clusters A and B. (**F**) Heatmaps of marker gene expression. (**G**) PCA analysis. (**H**) Immune cell fraction between clusters A and B. (**I**) Correlation analysis of marker genes and immune cells. (**J**) Immune cell fraction of four marker genes between clusters A and B

We could get the position of 11 marker genes on the chromosome from Fig. 5A. Our next step was to examine the relationship between marker genes and the prognosis of patients with different subtypes of OLP patients using the “ConsensionClusterPlus” R package for consensus clustering analysis based on the expression levels of the marker genes. A consensus matrix, we believe, is a better visualization tool that can help assess cluster composition and size. When k = 2, the color-coded heat map of the consensus matrix showed high intra-group correlation and low inter-group correlation, which strongly suggests that it is very appropriate to classify OLP patients into two subtypes (Fig. 5B). The increasing trend of the CDF values with respect to the consensus index indicates the existence of an appropriate classification, and according to the CDF curve and the Delta area, when the cluster index “k” increases from 2 to 9, k = 2 proves to be the best point to obtain the maximum difference between clusters, so we divided the OLP patients into two subgroups (Fig. 5C-D).

Next, we further explored the metabolic differences between marker genes in clusters A (low risk cluster) and B(high risk cluster). Both box line plots and heatmaps clearly showed that the vast majority of marker genes had higher expression in cluster B(Fig. 5E-F).

The distribution of risk in different populations is often visualized by PCA. Patients in cluster A and cluster B had significant differences when compared to each other. (Fig. 5G). In this cohort, immunotherapy played an important role in treatment, so it is important to understand the distribution and correlation of the 23 infiltrating immune cells. We calculated the level of immune cell infiltration in both clusters by the ssGSEA algorithm. It was found that the infiltration level of most immune cells was higher in cluster B compared to cluster A (Fig. 5H). In order to investigate the potential mechanism of 11 marker genes affecting immune infiltration, we drew a heat map of marker genes-immune cell correlation and analyzed the infiltration levels of 11 marker genes in two clusters, and the heat map results showed that PLEK, ITGAL, SLC43A3, HLA-DQA2 showed the most significant positive correlation with immune cells (Fig. 5I ), PLEK, ITGAL, SLC43A3, and HLA-DQA2 showed higher levels of immune infiltration in cluster B (Fig. 5J), and the immune infiltration levels of the remaining genes were released as shown in Supplementary Fig. 4.

### WGCNA analysis identifies and characterizes modules associated with clinical features

**Fig. 6:**
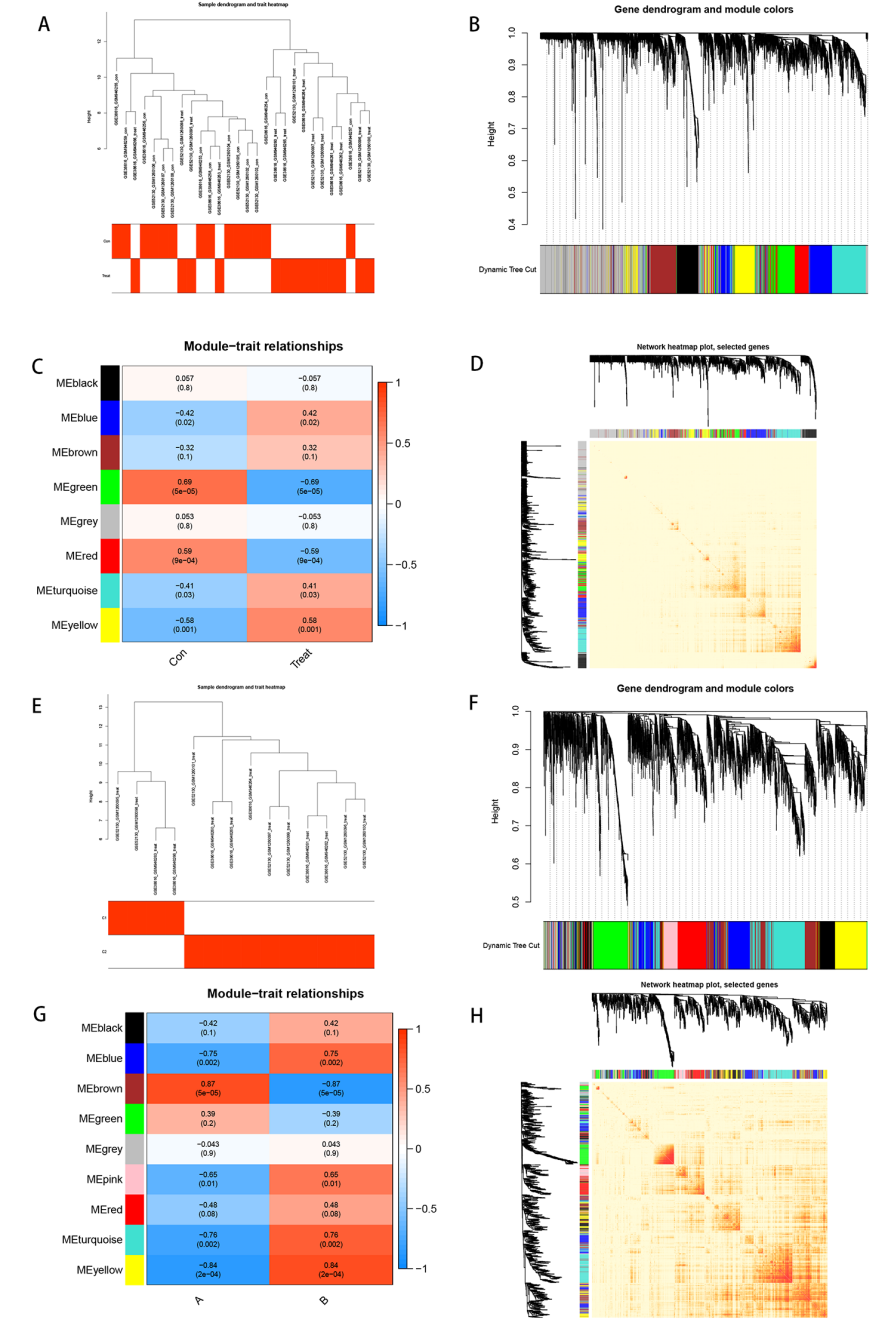
WGCNA analysis identifies and characterizes modules associated with clinical features. (**A**) Clustering dendrogram of GSE52130 and GSE38616. (**B**) Clustering dendrogram of genes, various colors represent different modules. (**C**) The relationship of clinical features and eight modules. (**D**) heatmap plot of Visualization of the WGCNA network. (**E**) Clustering dendrogram. (**F**) Clustering dendrogram of genes. (**G**) The relationship of OLP patient staging with nine modules. (**H**) heatmap plot of Visualization of the WGCNA network

Based on the WGCNA analysis, to explore the key gene clusters associated with the clinical traits of OLP, we first clustered the combined samples of GSE52130 and GSE28616 according to the clinical disease traits (normal samples, OLP samples) by using the spearman correlation coefficient method to obtain the sample clustering tree (Fig. 6A). Then a hierarchical clustering analysis was performed using the “WGCNA” R package to construct a gene co-expression network (Fig. 6B) with a soft threshold of 19 (R^2^ = 0.9) (Supplementary Fig. 2A) to obtain a scale-free network, and eight modules were identified after dynamic cropping. Each module has a different color, where the genes in the gray module are meaningless. The green module with the strongest and most significant correlation with clinical traits could be selected for subsequent analysis (Fig. 6C). Also based on WGCNA, we explored the gene clusters associated with OLP patient typing (A, B) and obtained a sample clustering tree after clustering the OLP samples (Fig. 6E) with a soft threshold set to 18 (R^2^ = 0.9) (Supplementary Fig. 2B). The gene co-expression network was obtained after further hierarchical clustering, nine modules related to typing were identified after dynamic cropping, and the brown module with the strongest and most significant correlation with typing was finally selected for subsequent analysis (Fig. 6F-G). To further examine the antagonism of genes between modules, we plotted the identified modules in the two TOMs in a heat map, in which genes with low overlap were shown in light color, while genes with high overlap will appear in dark red. Based on the results, we found that the genes in the 2 TOMs were relatively independent of each other (Fig. 6D, H).

### Enrichment analysis of genes related to the involvement of CD8 + cells in the pathogenesis of OLP

**Fig. 7:**
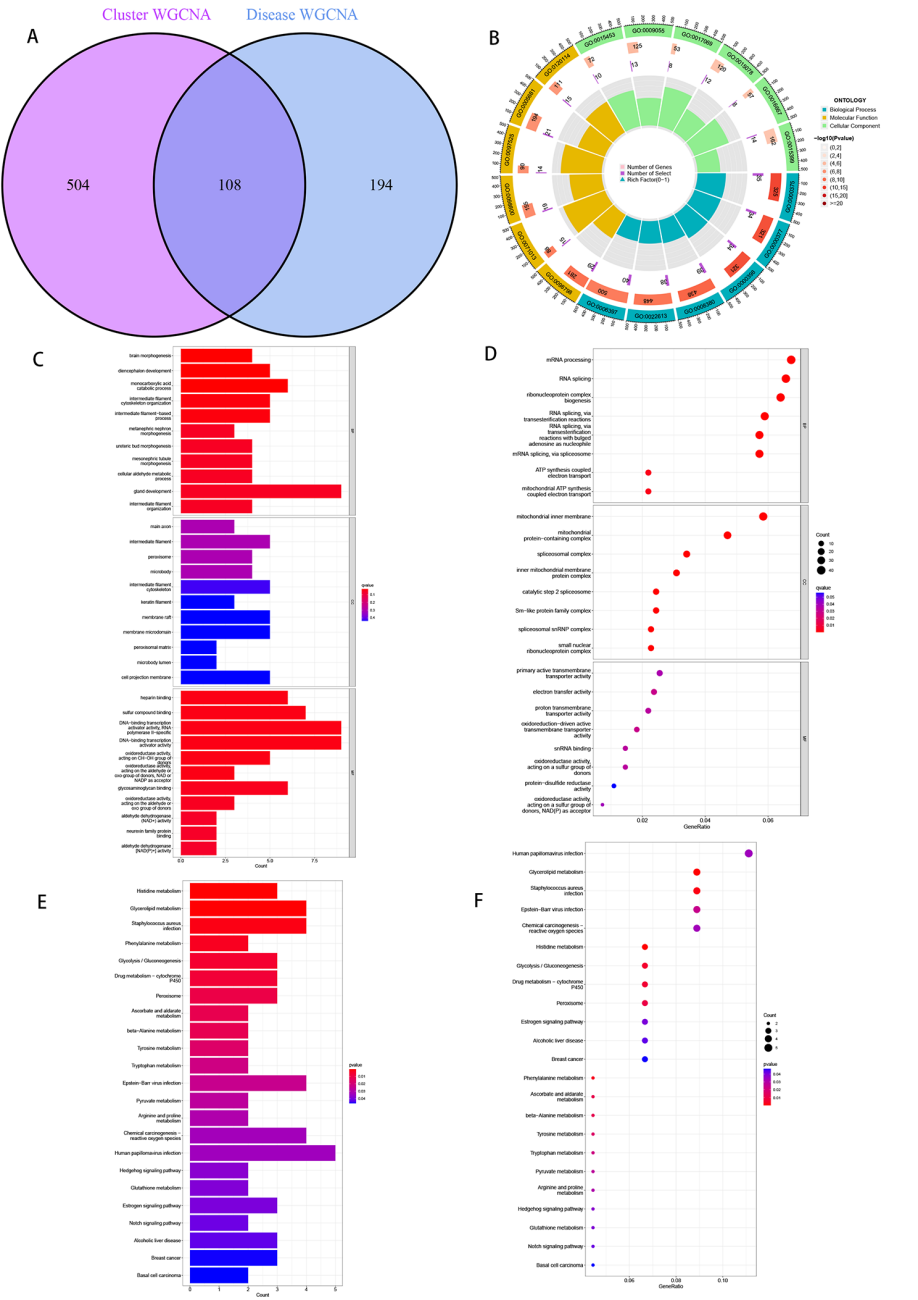
Intersecting genes were subjected to KEGG(www.kegg.jp/kegg/kegg1.html) enrichment analysis and GO functional analysis to assess their biological functions. (**A**) Cluster WGCNA intersected with disease WGCNA core genes. (**B-D**) GO analysis shows that many immune-related biological processes are enriched. (**E-F**) KEGG analysis shows that many immune-related pathways and pathogenesis-related mechanisms are enriched

The number of intersecting genes between 504 genes in Cluster WGCNAH and 194 genes in disease WGCNA was 108 (Fig. 7A). The threshold FDR < 0.05 and *P* < 0.05 were used to select the significantly enriched items. Biochemical processes (BP) are mainly involved in ATP synthesis and electron transport, RNA splicing via esterification reactions, and rRNA metabolism. Cellular components (CC) mainly include mitochondrial inner membrane protein complexes and oxidoreductase complexes. There are two main types of molecular functions (MF): redox-driven active transmembrane transporters and proton transmembrane transporters (Fig. 7B). GO analysis results proved that this crossover gene was mainly enriched in brain morphogenesis, and diencephalon development, (Fig. 7C ~ D). The kegg, on the other hand, proved that this crossover gene histidine metabolism, glycerolipid metabolism, in metabolic effects were significantly enriched (Fig. 7E ~ F).

### Construction of CMGs score

**Fig. 8:**
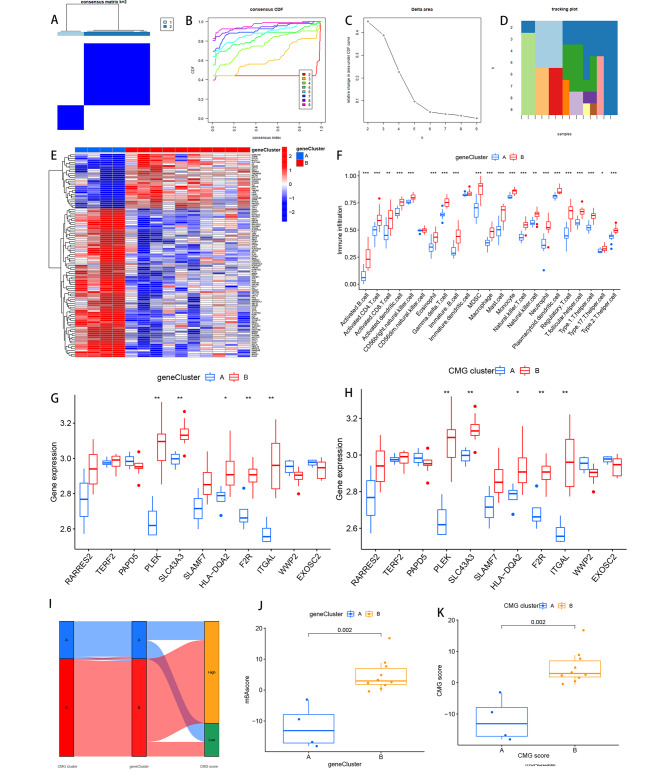
Consensus clustering identifies the molecular subtypes of intersecting genes. (**A**) Consensus matrix for k = 2. (**B**) Consensus clustering CDF for k = 2 to 9. (**C**) Relative change in area under the cumulative CDF curve for k = 2 to 9. (**D**) Tracking plot showing the number of consensus clusters for samples in each k. (**E**) Heatmaps of intersecting gene expression. (**F**) Immune cell fraction between cluster A and cluster B. (**G**) Immune cell fraction of tagged genes between gene clusters. (**H**) Immune cell fraction between CMGs clusters. (**I**) Sankey diagram of CMGs scores versus CMGs scores and gene scores. (**J**) Differences in CMGs scores by CMGs score type. (**K**) Differences in CMGs scores by gene score type

The optimal number of subtypes in OLP samples was determined as two using the “ConsensionClusterPlus” R package based on the expression of 108 genes (Fig. 8A-D). The differential gene expression in the two gene subtypes was indicated by this heatmaps (Fig. 8E). And the level of immune cell infiltration was analyzed by single sample gene set enrichment analysis (ssGSEA). The results revealed that the infiltration levels of most immune cells were higher in cluster B compared with cluster A, such as activated_B_cell, activated_CD4_T_cell, activated_CD8_T_cell, activated_dendritic_cell, MDSC macrophage, mast_cell, monocyte, and natural_killer_cell (Fig. 8F). Most marker genes were more highly expressed in the B isoform than in the A isoform in both genes cluster and CMGs cluster (Fig. 8G-H). The optimal number of subtypes in OLP samples was determined as two using the “ConsensionClusterPlus” R package based on the expression of 108 genes (Fig. 8I ~ K).

## Discussion

OLP is considered an inflammatory disease of the oral cavity mediated by immunity. However, it has been shown that 1.63% of OLP evolves into OSCC within 7 years and is therefore classified as a potentially malignant disease by WHO [26, 27].

The diagnosis of OLP is usually based on characteristic clinical features, It is usually easy to diagnose reticular OLP due to its specific clinical characteristics, including bilateral symmetrical transverse hyperkeratosis and low malignant potential [28]. However, other types of OLP such as atrophic, maculopapular, and erosive require histopathological diagnosis in most cases to clarify the clinical diagnosis and determine Whether there is abnormal cell proliferation, heterogeneity, and other signs associated with malignancy [29]. There is an urgent need for a non-invasive diagnostic method that can replace biopsy and help physicians accurately assess the patient’s condition. Numerous studies have shown that OLP is the result of the involvement and interaction of multiple immune cells, such as various subtypes of T cells, NK cells, mast cells, macrophages, etc. [30]. High-throughput sequencing excels at describing the overall disease picture and can demonstrate and identify at a very high level the disease/normal tissue gene expression differences [31]. but unfortunately, the role of different cell subpopulations in diseased tissues, the proportion of different cellular components within tissues, and cellular interactions are difficult to determine using bulk sequencing techniques [32]. single-cell technologies show great advantages in analyzing the cellular composition and cellular communication in diseased tissues [33]. in addition to this, the analysis of individual cells was temporally sequenced, thereby reconstructing pseudo-time-series and mimicking the real time-varying trajectory as closely as possible [34]. It is exciting to be able to reveal cell composition at different time cross-sections based on this approach and provide new perspectives for the exploration of mechanisms involved in disease progression. As a result of single-cell sequencing, we identified CD8 + T cells as the core cells of OLP.

It has now been shown that antigen presentation by basal keratin-forming cells and the killing of antigen-specific keratin-forming cells by CD8 + T cells are key aspects of OLP [35]. In addition, it has been found that T cells are major immune contributors to OLP and that activated CD8 + T cells are primarily found in the epithelium and near damaged basal keratin-forming cells [36]. In addition, CD8 + T cells secrete tumor necrosis factor-α and granzyme B, and CD95L on the cell surface binds to CD95 on the surface of keratin-forming cells, thus triggering apoptosis of keratin-forming cells and destruction of the epithelial basement membrane [37, 38]. In parallel, CD8 + T cells can release chemokines that attract other lymphocytes and immune cells to the lesion site, thereby promoting the development of OLP and the formation of an inflammatory environment [39, 40]. Overall, CD8 + T cells play a central role in the development of OLP and are extensively involved.

In order to classify patients into types A and B, we performed a clustering analysis using CD8 + T cell maker genes. As a result, there was a substantial difference in immune cell infiltration between the A and B types, with A types infiltrating significantly fewer immune cells than B types. It has been shown that in OLP, CD4 + helper T cells are the major cells of the lamina propria and are activated due to an increase in Langerhans cells and secretion of large amounts of IL-12. In the subepithelium and lamina propria, CD4 + T cells are the predominant lymphocyte population, whereas in the intra-epithelial lesions of OLP, activated CD8 + lymphocytes constitute the majority of infiltrating lymphocytes. It has been shown that mast cells in the OLP increase in number, degranulate, and secrete large amounts of inflammatory mediators thereby promoting lymphocyte exudation and adhesion [40–43].

To further investigate the linkage and mechanism of action between CD8 + T-cell marker genes and OLP, WGCNA screening of core genes was performed between AB subtypes and between diseased and normal groups, respectively, and then the results were intersected. Finally, 108 genes that were both CD8 + T cell-associated signature genes and OLP core genes were selected, an expression matrix was constructed and patients were classified into A and B types. The immune infiltration profile was found to be similar to the former while providing good discrimination between OLP patients, which is beneficial for dentists to accurately diagnose and manage OLP patients.

Undeniably this study has some limitations. Since the results were based on the analysis of public databases, there is still potential bias in this study, which may lead to inconsistency between the predicted and actual results. More clinical single-cell data from OLP patients and high-throughput sequencing data need to be collected in the future. The study should also be validated by prospective studies and basic experiments to improve the accuracy in practice.

## Electronic supplementary material

Below is the link to the electronic supplementary material.

Supplementary Material 1

Supplementary Material 2

Supplementary Material 3

Supplementary Material 4

## Data Availability

The datasets analyzed in the current study are available in the GEO (https://www.ncbi.nlm.nih.gov/geo/).
