# Supplementary figures and images for: CD8 + T-cell marker genes reveal different immune subtypes of oral lichen planus by integrating single-cell RNA-seq and bulk RNA-sequencing
Source: BMC Oral Health. 2023 Jul 8;23:464. doi: 10.1186/s12903-023-03138-0 (PMC10329325; doi:10.1186/s12903-023-03138-0)

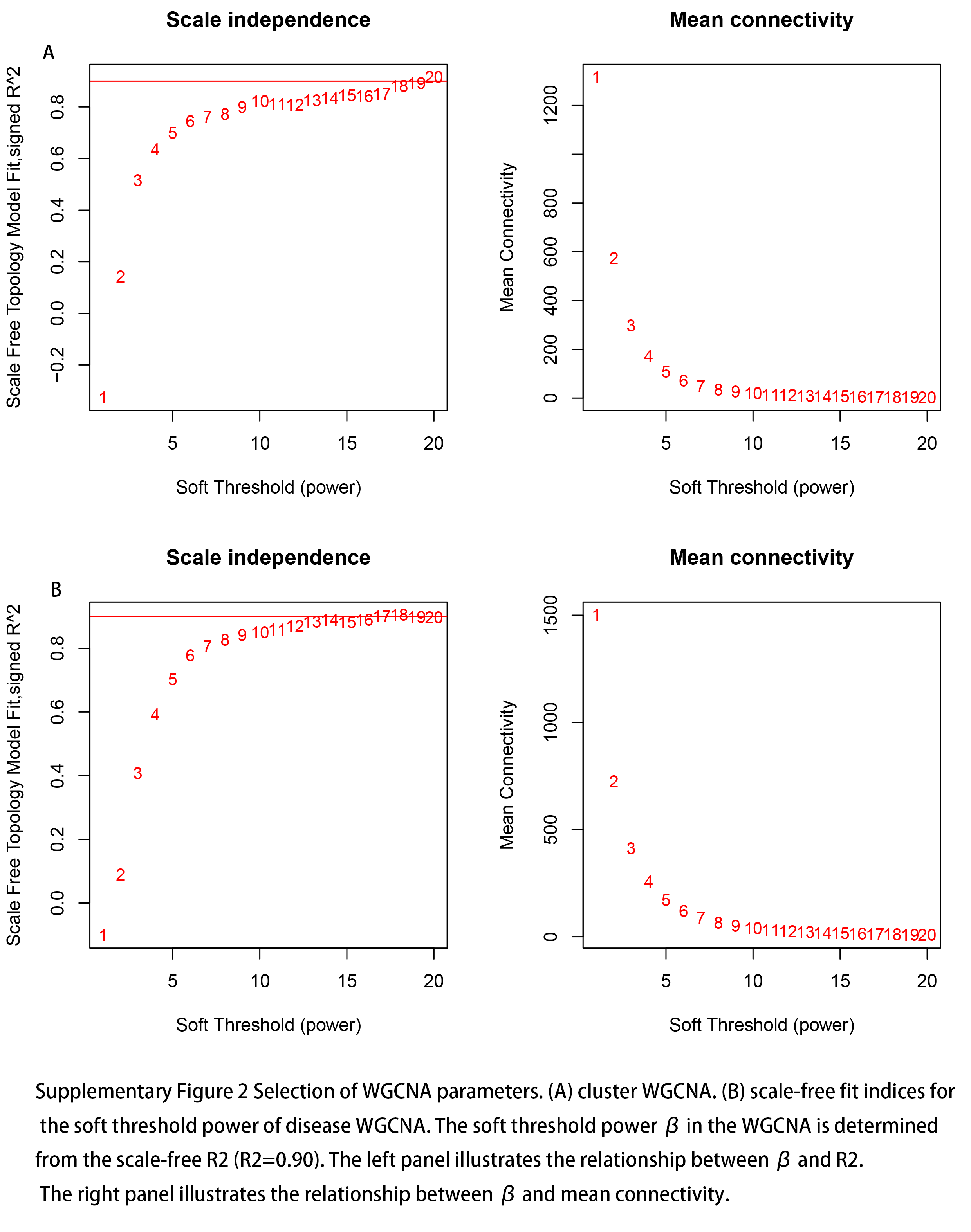

Supplement: Supplementary file 1 — Supplementary Material 1 [file 12903_2023_3138_MOESM1_ESM.png]

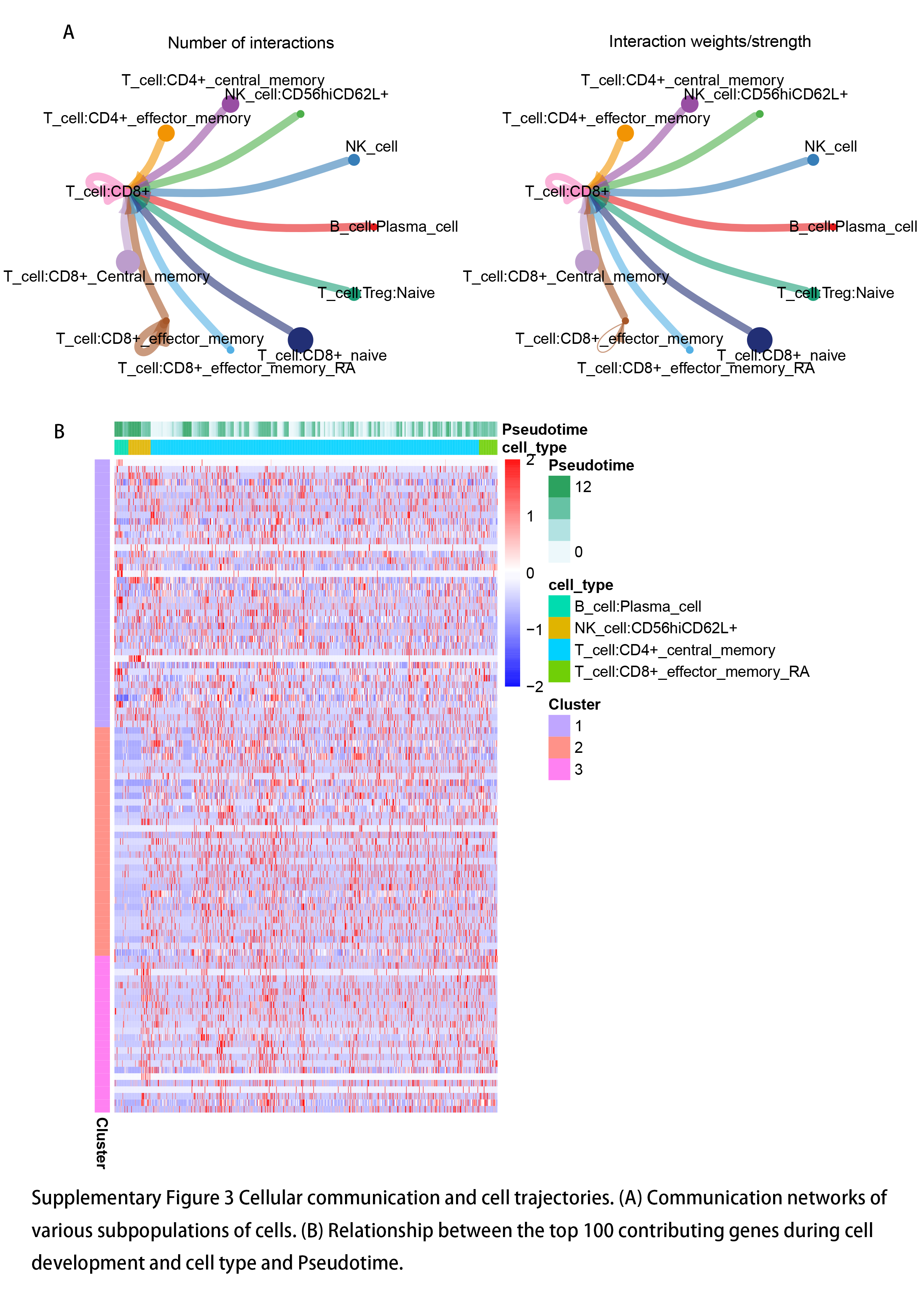

Supplement: Supplementary file 2 — Supplementary Material 2 [file 12903_2023_3138_MOESM2_ESM.png]

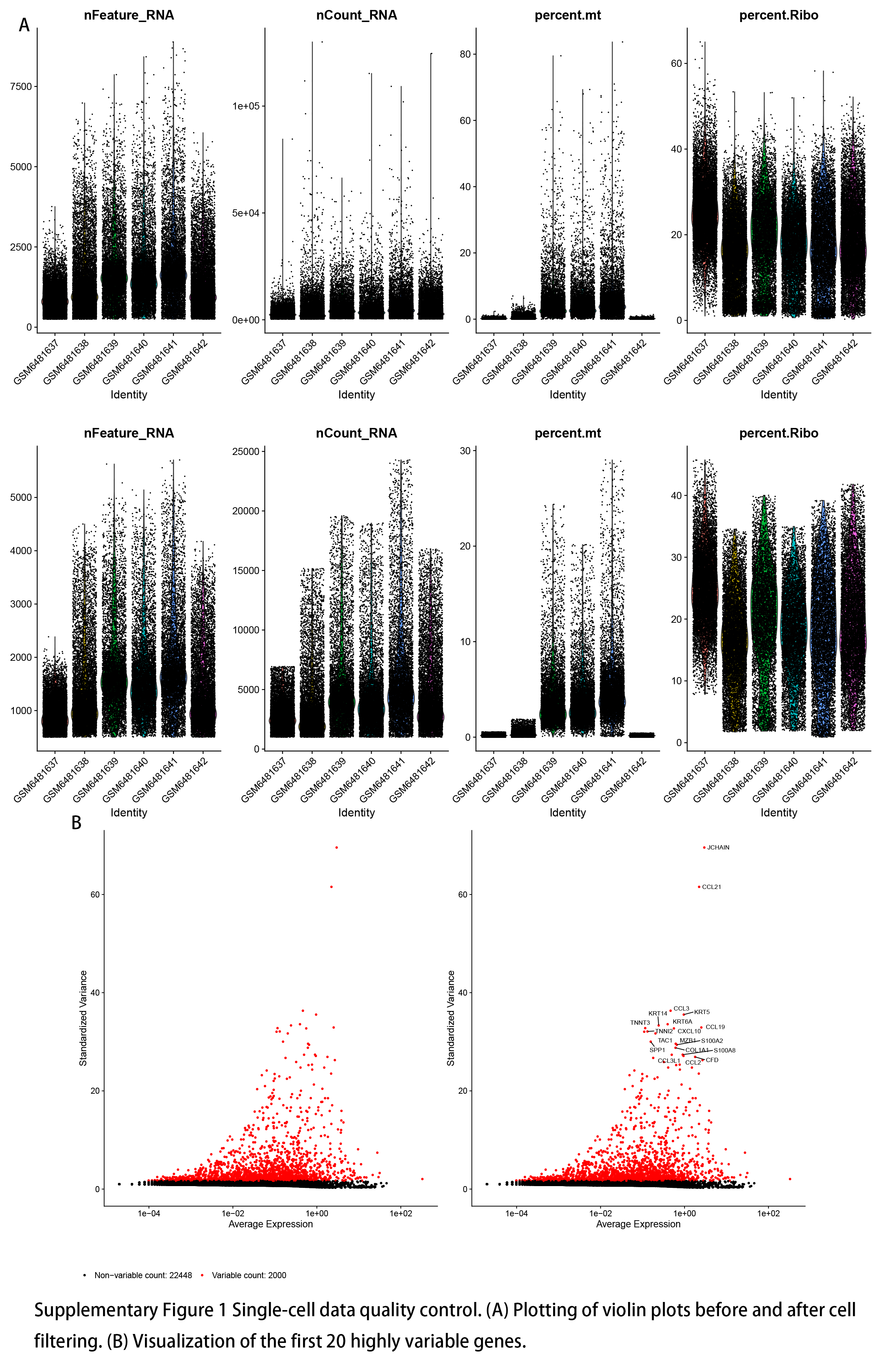

Supplement: Supplementary file 3 — Supplementary Material 3 [file 12903_2023_3138_MOESM3_ESM.png]

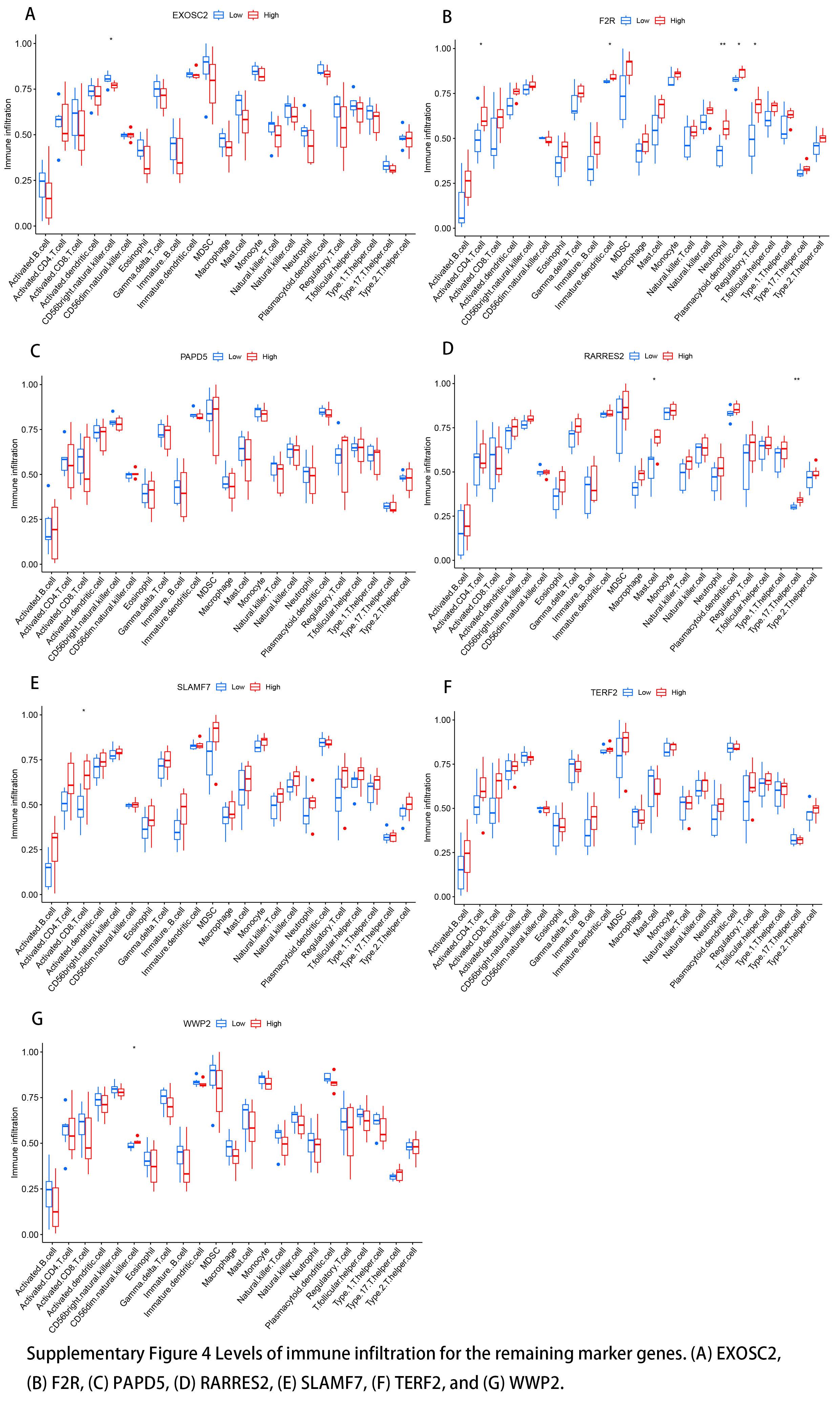

Supplement: Supplementary file 4 — Supplementary Material 4 [file 12903_2023_3138_MOESM4_ESM.png]
